# Unique supramolecular assembly through Langmuir – Blodgett (LB) technique

**DOI:** 10.1016/j.heliyon.2018.e01038

**Published:** 2018-12-17

**Authors:** Syed Arshad Hussain, Bapi Dey, D. Bhattacharjee, N. Mehta

**Affiliations:** a Thin Film and Nanoscience Laboratory, Department of Physics, Tripura University, Suryamaninagar 799022, Tripura, India; b Physics Department, Institute of Science, Banaras Hindu University, Varanasi 221005, India

**Keywords:** Nanotechnology

## Abstract

The Langmuir-Blodgett (LB) technique is a way of making supra-molecular assembly in ultrathin films with a controlled layered structure and crystal parameter, which have many envisioned technological applications for optical and molecular electronic devices as well as signal processing and transformation. Probably LB technique is the best method to manipulate materials at molecular level and provides a scope to realize the molecular electronics in reality. In this review article, we have discussed about the general introduction of LB technique and recent development on LB and related system including (i) LB methodology, (ii) characterizations of LB films, (iii) LB films and molecular electronics, (iv) historical review of LB films, (v) research and applications including fundamental research and application towards devices.

## 1 Introduction

Recently thin film science have attracted a great attention due to their many practical and commercial applications in various fields, such as sensors [7, 8], detectors [1, 2, 3, 4, 5, 6], surface coating [1, 2, 3, 4, 5, 6], optical signal processing [9, 10, 11], switching devices [12, 13, 14], molecular electronic devices [15, 16, 17, 18, 19, 20], nonlinear optics and models mimicking biological membranes [21, 22, 23], nanoarchitectonics as well as molecular machine [24, 25, 26]. Generally, the basic requirement for such applications are,- a perfectly organized molecular thin films having specific properties, careful alignment of the molecules and a substrate which have very good stability with respect to thermal and chemical changes. Langmuir-Blodgett (LB) technique is a powerful tool in creating carefully controlled supramolecular structures of organized molecular assemblies [27, 28, 29, 30, 31, 32], which have their potential applications in above mentioned areas. By changing different film forming parameters during LB film formation, it is possible to control the bulk properties of the materials that were incorporated into the LB films. Due to the tremendous advancement of preparation and characterization tools it is possible to synthesize organic molecules having any desired structure and functionality without any limitation. In addition to that flexibility and over all control on the film structure prepared using LB technique enables the production at electrically, optically and biologically active components on a nanometre scale. Perhaps the LB technique is the most suitable method for the realization of nano to micro order thin films of organized system with wide range of application potential [5]. In this paper an overview of the LB technique and its application potential have been reviewed.

## 2 Main text

### 2.1 Langmuir–Blodgett (LB) methodology

The Langmuir – Blodgett (LB) technique offers the possibility to obtain highly ordered well defined and controlled mono/multilayers and realizes the construction of ultimate molecular architectures which allow the study of physical phenomenon on a molecular level.

**Fig. 1:**
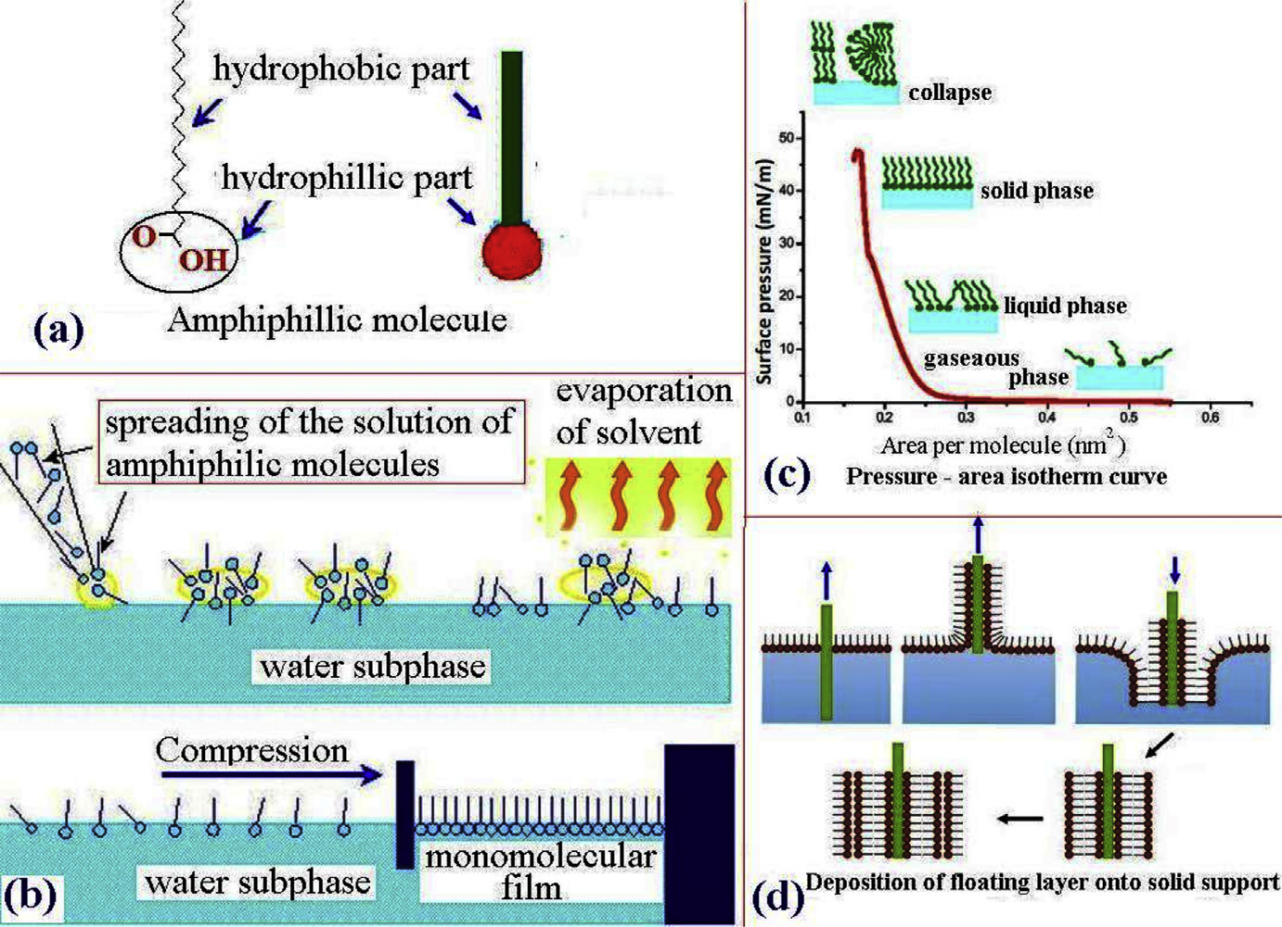
Langmuir – Blodgett (LB) technique.

Typically LB compatible materials are amphiphilic molecules having two distinct regions, which are a hydrophilic head group (water loving) and a hydrophobic tail group (water hating) as shown in Fig. 1a. They must be soluble in organic non polar and water immiscible solvents. Water insoluble amphiphilic molecules form floating monolayer at air-water interface. Long chain fatty acid, lipid molecules etc are the example of LB compatible materials.

In principal LB technique consists of two main steps-

(a) Preparation of floating monolayer film at air-water interface (Langmuir-film)
(b) Deposition of the Langmuir film on solid substrate (LB film)

Initially selected amphiphilic materials is dissolved in a volatile and water in soluble solvent (chloroform, benzene etc)to prepare dilute solution. After that minute amount of the solution is spread onto air-water interface at the LB trough using a micro syringe. Spreading should be done from very close to the water surface so that the solution and hence the molecule remain at air-water interface and do not dip into the water. Soon after the spreading the molecules spread through out the interface (available area) on the trough. After the evaporation of solvent almost one molecule thick layer of the amphiphile remained at the interface, with the head groups immersed in the water and the tail group lying outside (Fig. 1b). Normally the amount of molecules (concentration) is kept as low as possible so that initially the molecules remains far from each other and exert very little force on each other. Under these circumstances, the interaction between the molecules is very low (lateral adhesion is almost zero) and the resulting floating monolayer can be considered as a two – dimensional gas due to the large inter molecular distances (Fig. 1b & C).

Idea about the surface behaviour as well as stability of the floating monolayer has been estimated by recording surface tension. Surface tension is affected to a large extent due to the presence of molecules at air-water interface. Initially the effect of spread molecules on the surface tension is relatively low. When the computer controlled barrier is minutely compressed, the available surface area of the floating Langmuir film is reduced. As a result inter molecular distance between the adjacent molecules decreases and the surface tension decrease further. Amphiphilic molecules in the floating Langmuir film start to interact with each other. The force exerted by the floating film per unit length can be considered as the two dimensional analogue of pressure, known as the surface pressure (π).

Typically, this surface pressure (π) is equal to the decrease in surface tension of air – water interface due to the presence of monomolecular film and can be expressed as:

$$\pi =\gamma_{0}-\gamma$$

where $\gamma_{0}$ is the surface tension of the empty air – water interface and γ the surface tension of the same in the presence of a monolayer.

During the compression of the monolayer, self organization of the molecules occur During the self organization process floating monolayer at the interface under goes several phase changes very similar to the three dimensional gaseous, liquid and solid states (Fig. 1c). In solid state the molecules finally form a compact and well ordered two dimensional film at air – water interface (Fig. 1b). These changes in the monolayer phases during compression are monitored by measuring the surface pressure – area per molecule (π –A) isotherm. During the self organization process floating monolayer at the interface under goes several phase changes very similar to the three dimensional gaseous, liquid and solid states (Fig. 1c). Schematic of a typical π –A isotherm curve is shown in Fig. 1c. In LB technique the π –A isotherm can be considered as two dimensional finger print to have idea about the thermodynamic behaviour of the floating monolayer [33, 34].

It may be noted in this regard that the shape, nature of the isotherm curve as well as the monolayer phases are largely depend on the condition of subphase-pH, temperature and also on the hydrocarbon chain length and head group of the spread molecule [35]. Factor governing the monolayer phase behaviour have been listed in Table 1. As a whole π-A isotherm provides information about thermodynamic behaviour and stability of the floating film, the reorientation of the molecules and existence of the phase transition as well as conformational transformation of air-water interface [34]. For further discussions on pressure – area isotherms, the reader can refer to different books and reviews dedicated to Langmuir and Langmuir–Blodgett films [34, 35, 36].

**Table 1:** Factors governing the monolayer phase behavior.

**Van der Waals interaction:** • Short range forces: (Γ_VDW_ ∝ r^−6^) • Directly proportional to the number of carbon atoms in the hydrocarbon chain for the saturated hydrocarbon • Depends on the presence of double bonds or substituents like methyl group in the chain
**Electrostatic interaction:** • Ion – Ion (Γ_ii_ ∝ r^−1^) • Ion – Dipole (Γ_id_ ∝ r^−2^) • Dipole – Dipole (Γ_dd_ ∝ r^−3^)
**The subphase composition:** • pH of the subphase • Temperature of the subphase • Type/nature of materials

Langmuir monolayer floating at air-water interface can be transferred onto solid substrate to have mono or multilayer Langmuir-Blodgett (LB) films. Typical example of substrate commonly used for LB film deposition are listed in Table 2. During deposition the surface pressure is kept fixed at a desired constant value. Deposition of Langmuir monolayer onto solid substrate can be done in a number of ways – X-type, Y- type, Z-type deposition [34, 36]. Depending on the nature of the substrate is whether hydrophilic or hydrophobic the first layer (Langmuir film) will be transferred onto the substrate either when raised or lowered through the interfacial film. Subsequently, successive depositions of single layers occurred during each traversal of the substrate through the monolayer film at air – water interface. Such a deposition mode is called vertical deposition or Y-type deposition. Multilayer film can be deposited through repeated lowering and raising of substrate through the floating layer. In Y-type deposition film possess head-to-head and tail-to-tail configuration [34]. Y type deposition is very often used in LB technique. However, in certain cases the floating monolayer is transferred only during the immersion (X-Type deposition) or the emersion (Z-type deposition) of the substrate. LB film deposition typically depends on the pH & temperature of the subphase, humidity, deposition speed, deposition pressure, layer number and contact angle between the substrate and floating layer during deposition [37, 38, 39, 40, 41, 42, 43].

**Table 2:** Substrates commonly used for LB film deposition.

Monolayer can be transferred to many different substrates according to need viz – • Glass, quartz, mica etc • Aluminum, tin and their oxide • Silicon wafer, Gallium arsenide wafer • Gold, Silver • Cleaning of substrates is very important

Ideally the LB technique offers the possibility to control and manipulate each step of the LB film preparation precisely. The main advantage of LB deposition is the attainment of a molecular arrangement perfectly organized at the air – water interface, which can be maintained during the transfer onto the solid support by optimizing all the parameters. Importance of the LB film studies have been listed in Table 3.

**Table 3:** Importance of LB film studies.

• Uniform deposition of the monolayer over large areas • Multilayer structures with varying layer composition • Assembly of individual molecules into 2D & 3D systems • Hetero structured multilayer film consisting of various molecules • Control of film structure & monolayer thickness at the molecular level • Almost any kind of solid substrate can be used • Unique blend of Physics, chemistry & molecular engineering • Understanding of structure - property relations

### 2.2 Characterizations of Langmuir–Blodgett (LB) films

Characterizations of molecules assembled onto thin films are very crucial to have idea about the utility of such films. Due to tremendous advancement in the characterization techniques it is possible to visualize as well as manipulate different materials at nanoscale dimension. Different characteristic tools have been used to study the LB film properties based on the application perspective. Few commonly used techniques have been listed in Table 4 below,-

**Table 4:** Few important techniques commonly used to characterize LB films.

Experimental technique	Information extracted
Brewster Angle Microscopy (BAM)	In-situ study of various phases of the thin films at interfaces. It is sensitive to the surface density and to the anisotropy of domains in monolayer.
Fluorescence Imaging Microscopy (FIM)	In-situ domain structure study (micrometer range)
Surface potential measurement	Polarization, orientation.
Infrared reflection absorption spectroscopy, Attenuated total reflection – Fourier transform infrared (ATR-FTIR)	Hydrocarbon chain packing & conformation, degree of ionization of the head groups, H-bonding, chemical and structural changes, molecular orientation.
Ellipsometry	Refractive index & thickness measurement (∼2A^0^)
X-ray diffraction/reflection Grazing incidence and small angle – X-ray diffraction	Inter layer spacing, in-plane lattice structure In-plane/out-of-plane orientation/tilt of hydrocarbon chain.
Neutron diffraction	Inter layer spacing.
UV-Vis absorption spectroscopy	Electronic transition & orientation.
Raman Spectroscopy & Surface-enhanced Raman Scattering/Spectroscopy (SERS)	Identification & orientation, conformation of alkyl chains and head groups, molecular interactions within LB films.
Optical harmonic generation (2^nd^ & 3^rd^ order)	Non-linear coefficient, orientation.
Optical microscopy Confocal fluorescence	In-plane structural information. Molecule orientation, lipid domain morphology, grain boundaries, microcollapsed region (lateral resolution of 0.1 μm).
Scanning electron microscopy (SEM) Transmission electron microscopy (TEM)	Surface morphology, domain structure, patterns, pinholes and defects (in-homogeneous crystalline domains, micro-collapse etc) (resolution of 50 nm).
Scanning tunnelling microscopy (STM) Atomic force microscopy (AFM)	Imaging surface in the atomic level, visualisation of defects (grains, pinholes, lateral heterogeneity, disclinations etc), sub-molecular packing (lateral resolution of 0.2 nm).
X-ray photoelectron spectroscopy (XPS) or Electron spectroscopy for chemical analysis (ESCA)	Quantitative analysis of chemical composition of the film surface.
I – V, C – V, resistivity etc measurement	Electrical behaviour.
For further details, the reader can refer to different related books and review papers [34, 35, 36, 50, 51, 52, 53, 54, 55, 56, 57, 58, 59, 60, 61, 62, 63, 64, 65, 66, 67, 68, 69, 70].	

### 2.3 Langmuir – Blodgett (LB) films and molecular electronics

In molecular electronics a single molecule or a group of molecules arranged in a desired manner act as the basic building blocks of electronic devices. Thin films of desired material are very crucial in this regard. Out of various existing thin film preparation techniques Langmuir-Blodgett (LB) technique is one of the best suitable technique to organize molecules onto thin films in a desired fashion with molecular level control. Specific advantages of LB technique have been listed in table – 3 of Section 2 in this manuscript. Uniqueness of the LB technique is that it enables us to construct functional two dimensional (2D) material with controllable and scalable growth. Here the organization can be manipulated by controlling various LB parameters viz – speed, composition of mixing, ph and temperature of subphase, changing deposition scheme etc. Thus LB technique provide a way to connect the macroscopic action to control/manipulation at molecular scale. Therefore, it can be said that LB technique may play a vital role to realize the dream of molecular electronics in reality. Till to date LB films have been used in many practical applications including microelectronic and opto-electronic devices, biosensing as well as chemical sensing, lithography etc [34, 36]. The following sections of this article describe some typical applications involving LB films.

### 2.4 History of Langmuir – Blodgett films

In the early history, it was noticed that oil forms a thin layer over water surface when spread onto the water surface which showed the properties like the dampening surface waves and ripples. The scientific breakthrough in the era came into notice when in 1774 Sir Benjamin Franklin, an American Scientist first reported to the British Royal Society, the calming behaviour of a teaspoonful of oil (2 ml) over an acre of a pond on Clapham [44]. It was Lord Rayleigh [45] who first gave the idea about the thickness of such floating oil films. He commented that maximum thickness of this oil film is one molecule thick. Agnes Pockel demonstrated that this oil film could be controlled by movable barrier [46]. Based on Pockel's experiment modern Langmuir trough has been developed. Extensive monolayer theory was developed by I. Langmuir [47]. He was awarded Nobel prize for his experiments suggesting that single layers of atoms that were bound within the underlying surface could be formed an adsorbed films of great stability. However, several years later it was K. Blodgett who for the first time developed a technique for transfer of the floating films from the air-water interface onto solid substrate [48]. Such transferred mono- and multilayer assemblies on solid substrate are now referred to as Langmuir-Blodgett (LB) films. After the pioneering work done by Langmuir and Blodgett, it took almost half a century before scientists all around the world started to realize the opportunities of this unique technique. It was Khun and his collaborators who revived and demonstrated the potential of the field [49]. His work stimulated interest in the topic in Europe, USA and Japan and now throughout the world. The first international conference on Langmuir-Blodgett (LB) films was held in 1979 and since then the use of this technique is increasing among the scientists throughout the world working on various different field of research. Extensive studies on LB films are now being going on. Various review articles has been published time to time since the revival of the interest in research related to different aspects of LB films [50, 51, 52, 53, 54, 55, 56, 57, 58, 59, 60, 61, 62, 63, 64, 65, 66, 67, 68, 69, 70].

### 2.5 Research and applications

Extensive research work on various aspects of LB films are going on throughout the world. In broad sense recent studies in the field of LB films research can be largely divided into two areas – (i) fundamental studies related to Langmuir and LB films and (ii) applications towards passive and active components of devices.

#### 2.5.1 Fundamental research

Since the discovery, LB technique has been used as an ideal method for a broad range of scientific research [2, 50, 51, 52, 53, 54, 55, 56, 57]. Most of the recent research work in the area of Langmuir and LB films are inspired by the pioneering research work done by H. Kuhn [71, 72]. It is the Kuhn, who demonstrated that LB technique can be used to manipulate the organization of functional molecule with in complex assemblies in a desired manner.

**Fig. 2:**
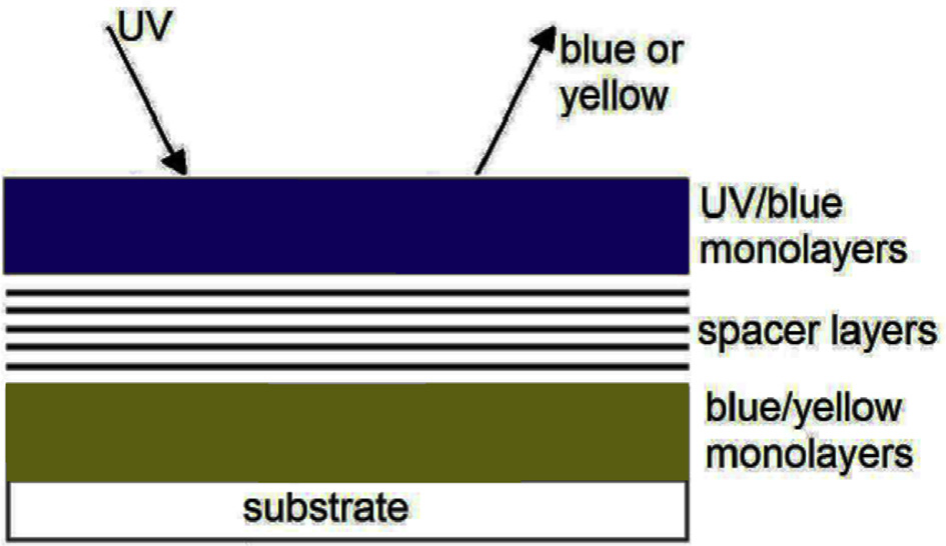
Kuhn's experiment of two blocks of different fluorescent layers, separated by a block of normal “spacer” layers.

One example from Kuhn's work is shown in Fig. 2. A block of fluorescent LB films that absorbs blue light and emitting in the yellow is separated from another block of different fluorescent LB films that absorbs in the UV range and emitting in the blue, by a block of fatty acids (spacer layer). He demonstrated that assuming a UV-light incident on the structure, it's possible to have either blue emission or yellow emission from the structure. The overall emission of the structure has thus changed from blue to yellow when the block of spacer layers is thin enough. Here energy transfer between the layers occurred due to the quantum mechanical tunneling, where the spacer layers can control the energy transfer process.

LB technique can be used to mimic natural biomembrane artificially [59, 65, 66, 73, 74]. Accordingly LB system has been used in biological research in a variety of ways [58, 67, 69, 75, 76]. LB films of certain materials have been used to immobilize an enzyme or a protein, which can trap and bind with some ions or molecules that change some measureable property of the film [59, 65, 67]. Accordingly the properties of those enzymes and proteins can be studied [77, 78, 79]. Recently LB technique has been used to investigate the lipid layers containing artificial tears [80]. Lipid-containing artificial tears are used to restore the lipid layer in thin films.

In case of urinary stones calcium oxalate monohydrate (COM) biomineral is a major component. In order to prevention or treatment of urinary stone disease it is essential to understand the processes which lead to COM precipitation in the urinary tract. For the studies of COM precipitation and adhesion, LB technique has been used to prepare phospholipid model domains in biological membranes. The effect of chemical and organizational properties of the model membrane towards the formation of COM crystals at the membrane interface can be explored from this study [81, 82]. Lots of efforts have been given to investigate different kinds of molecular aggregate formed onto LB films [37, 38, 39, 40, 41]. It has been shown that this technique is very suitable for preparation of different supramolecular aggregates, where the properties of such aggregates can be tuned by optimizing various parameters during film formation [83, 84].

#### 2.5.2 Applications towards passive and active components of devices

The most common application of LB films as the passive layers in several areas of commercial interest includes lithographic photoresist [85, 86, 87, 88], lubrication [89, 90, 91] as well as enhancing device performances such as surface acoustic waves [5, 90, 92, 93, 94], liquid crystal alignment [77, 95, 96, 97] etc. For creating devices with smaller dimensions the electron-beam lithography is used. It requires a very thin resist for enhancement of resolution due to the pronounced scattering in a resist of normal thickness. LB films have been used as photoresist viz, LB films of fatty acid salts as a positive resist and of the 22-tricosenoic acid as a negative resist have been used with good results [85, 86, 87, 88]. Langmuir-Blodgett films have been used as lubricating layers for enhancing the useful life of high density hard disks [89]. LB films have also been used for the lubrication of magnetic tapes [91]. The friction coefficient and wear of the tape has been shown to dramatically reduced by coating the tape with seven layers of barium stearate.

The most significant passive application of LB films could however be for the alignment of liquid crystals. The dipole energy of the underlying substrate mainly governs the alignment of liquid crystals. By tailoring the structure of LB films it is possible to control the dipole energy and hence the orientation of liquid crystal. It has been shown that some liquid crystal molecules align spontaneously when sandwiched between two glass plates covered with a single layer of a polymer LB film [77, 95]. Due to its various attractive features acousto-electric device can be used to design physical and chemical sensors. Surface acoustic wave (SAW) oscillators have very high sensitivity and are widely used. LB technique has been employed to prepare gas absorbent layers on the surface acoustic wave oscillators [93, 94].

Pyroelectric materials can be used as detectors of infrared radiation. In this regard pyroelectric materials are advantages compare to narrow band-gap semiconductors. The main advantages of LB technique in the application of pyroelectric devices are, - (i) here symmetry of the film structure can be precisely manipulated and controlled through sequential deposition of monolayer. Also monolayers of different materials can be deposited to produced highly ordered heterostructure. (ii) The polarization of an LB film is ‘frozen-in’ during deposition and it is therefore not necessary to subject the film to a poling process. (iii) Amphiphilic organic molecules have low permitivities (ε) and LB films are ingeneral prepared using water insoluble amphiphilies. Accordingly the figure of merit for voltage responsivity pε (where p is the pyroelectric coefficient) is expected to be large. Finally the LB method enables the preparation of much thinner films than usually attained by other normal conventional techniques. The pyroelectric coefficients for multilayer acid/amine LB films can be about 10 $\mu Cm^{2}K^{-1}$ and depend on the thermal expansion coefficient of the substrate, indicating that there is a significant secondary contribution to the measured pyroelectric response [1, 36, 98, 99, 100]. LB films of poly(vinylidene fluoride trifluoroethylene) showed ferroelectric switching with distribution of switching times several decades wide [101].

Rectifying junctions are the oldest practical semiconductor device and are used as the basic elements of many electronic devices. The rectifying metal-semiconductor junction forms a schottky barier. LB film based polymer p-n junction as well as schottky junction have been investigated extensively [102]. Anion doped polypyrrole/polythophene in LB films have been found to behave as n-type semiconductor [103]. Various metals have been deposited onto cadmium stearate (CdSt_2_) LB films to have metal-insulator-semiconductor (MIS) structures [104]. LB films of ZnO has been used to fabricate metal-insulator-metal tunnel diode [105]. This types of diodes have great potential for use in infrared detection and energy harvesting applications. Polypyrrole and polythiophene derivatives assembled onto LB films have been investigated for realization of electrical rectifying devices [106]. Alternating deposition of this two polymers using LB technique have been done to fabricate heterostructure rectifying devices [107]. Aviram and Ratner predicated that molecule having a donor and an acceptor group connected by a short σ-bond should exhibit diode characteristics [108]. Based on their predication several researchers tried to realize LB film based rectifying diode [36]. It has been demonstrated that the rectification behaviour of such diode can be manipulated by changing LB film structure [109, 110].

Experimental and theoretical confirmation of anion-induced dipole reversal in cationic dyes due to molecular rectification have been demonstrated [111]. There is a good review article by R. M. Metzger on the application of Langmuir-Blodgett (LB) films as unimolecular rectifier [112, 113].

It has been observed that phthalocyanine derivatives show very good thermal and chemical stability when assembled onto LB films. Accordingly different phthalocyanine derivatives have been extensively investigated to realize LB film based field effect transistor (FET). Recently an interesting work about a physico-chemical investigation of carboxylic ionophores and phospholipids for application as ion selective field effect transistor (ISFET) has been reported [114].

Cui et al. [115] demonstrated depletion-mode n- channel organic field effect transistors (OFETs) based on naphthalene-tetracarboxylic-dianhydride (NTCDA) where, n-type NTCDA acts as active channel material due to its high mobility of 0.06 cm^2^V^−1^S^−1^, and p-type conducting polymer polypyrrole performs as the source and drain. Koezuka et al. [116] fabricated a FET by using two different kinds of conducting polymers, polypyrrole and polythiophene. Application of Langmuir-Blodgett films containing regioregular poly (3-hexylthiophene) polymer for application in Field-Effect Transistor (FET) have also been reported [117]. Thin-Film Transistors based on LB films of heteroleptic Bis(phthalocyanine) rare earth complexes has also been demonstrated [118]. LB films based Organic field-effect transistors for an extended porphyrin analogue – cyclo[6]pyrrole and neutral long-chain TCNQ derivatives have also been reported (list in Table 5) [119,120].

**Table 5:** Carrier mobilities of organic semiconductor based Field Effect Transistor [119, 120, 121].

Materials	Carrier Mobility (cm^2^V^−1^s^−1^)
Polythiphene	10^–5^
Polyacetylene	10^–4^
Phthalocyanine	10^−4^ – 10^−2^
Thiophene derivatives	10^−4^ – 10^−1^
Pentacene	10^−3^ – 3
C_60_	0.3
Organometallic dmit complex	10^–1^

Of late semiconducting organic films have been used to constract FET devices [121]. It has been observed that remarkable increase in carrier mobility occurred for the devices that has been built using LB films of organic materials during the last fifteen years [122, 123, 124, 125].

**Fig. 3:**
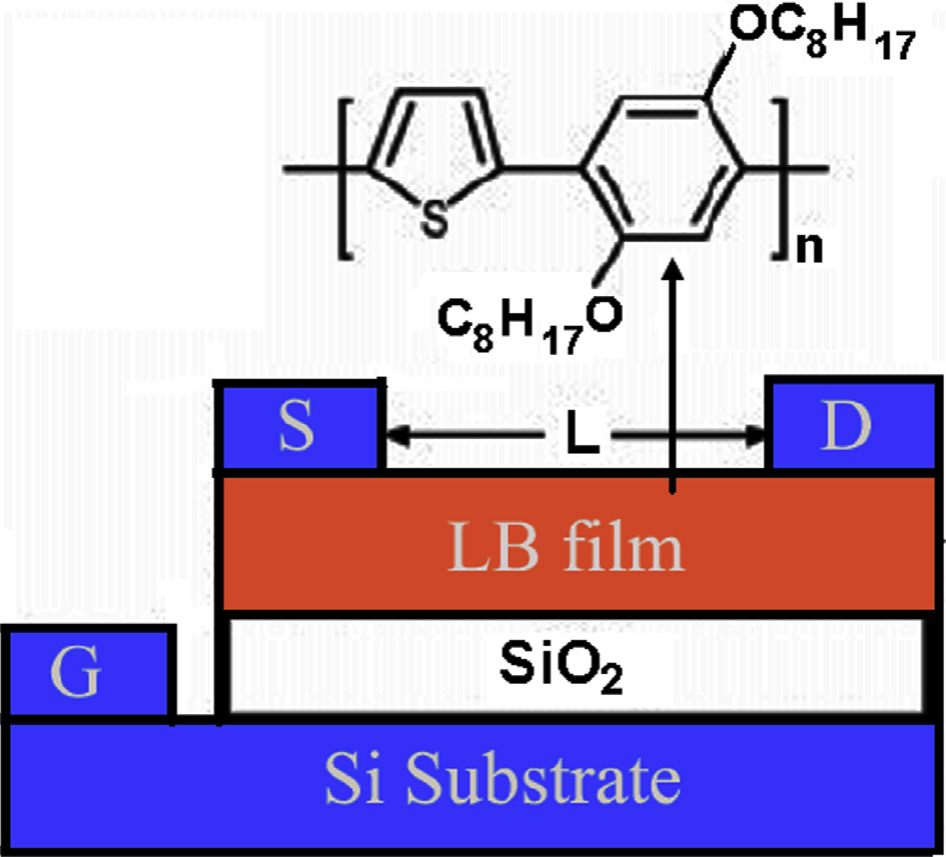
Schematic diagram of a LB film based TFT device. (Reprinted from Ref. [128], with permission from American Chemical Society).

Disiloxane based LB films showed high potential for OFETs applications for large-area organic electronics [126]. Large – area heterostructures consisting of silica and quantum dots deposited by LB technique, sandwiched between two grapheme sheets has been used for FET applications [127]. This type of heterostructures paves the way for developing novel hybrid opto-electronic devices through integration of 0D and 2D materials using LB method. Langmuir-Schäfer (LS) and cast films of poly (2,5-dioctyloxy-1,4- phenylene-alt-2,5-thienylene) have been used as the active layers to design LB film based transistor devices [128]. Interestingly LS thin film based device showed a field-effect mobility of the order of 5 × 10^−4^ cm^2^/V.s Although cast film based device did not show any such transistor behavior. This suggested that LB technique provided a favorable condition with respect to molecular organization in thin film making the system suitable for electron transport. A schematic diagram of LB film based TFT device is shown in Fig. 3.

After the discovery of electroluminescence in thin organic polymer layer, researchers throughout the world showed much interest in polymer light emitting diodes (PLEDs) [129, 130]. Conducting polymers have found potential application as luminescent materials which can replace the conventional inorganic light emitting materials for use in large area, light weight, flexible displays. These materials has attracted much attention due to their distinctive advantages over conventional luminescent materials such as,- low operating voltage, tuning of wavelength emitted by chemical modification, flexibility, low cost, easy processing, possibility of making large device and output colours in whole visible spectrum. Several p-doped conducting polymers have been tested in LB films [131, 132, 133, 134, 135] and have been used as hole injecting electrodes, like polypyrrole, polythiophene derivatives and polyaniline, which have high work functions, providing low barriers for hole injections.

LB film based light emitting device can utilize the ability of LB technique to organize the molecules in an ordered arrangement. For example, polarized light emission cab be achieved due to preferential alignment of molecules in LB films [136, 137]. LB technique offers the opportunity to manipulate the position and orientation of the luminescent species by controlling various LB parameters within metal mirror microcavities [138]. In order to enhance the lifetime of OLEDs several experiments on MEH-PPV LB films [139] have been done. Mixed LB films of pyridine and oxadiazole have been used which showed further increase in the electron-injection [139].

Many conducting polymers such as polyacetylene, polythiophene, polyindole, polypyrrole, polyaniline etc have been reported as electrode materials for rechargeable batteries [140]. Photoconductivity involves enhancement of the electrical conductivity of the material by the absorption of a suitable photon. It finds wide range of applications in electronics and opto-electronic devices. Polypyrrole LB films (band gap 3.2 eV) bassed devices is found to exhibit good photoconductivity [141]. Liu et al. [142] fabricated a polymer based capacitor, using polypyrrole and poly (3,4-ethylenedioxythiphene) poly(styrenesulfonate) as a semiconductor and gate layer. Dielectric polymer, polyvenylphenol, was applied as the insulator to the device. Composite electrodes for supercapacitors were prepared via chemical polymerization [138] of pyrrole on the surface of a porous graphite fiber matrix [143].

Nowadays, molecular electronics is an important emerging technology. It deals with the design, processing and device application of organic molecules at the molecular level/nanoscale level. LB technique is probably one of the best methods among various film forming techniques to realize organized molecular assemblies at nanoscale and may play a vital role towards molecular electronics. Resistive switching behaviour (bipolar and threshold) of organic molecules assembled onto LB films have been demonstrated successfully [84, 144]. It was possible to control the switching by optimizing the measurement protocol. This type of bipolar switching will play crucial role in future organic electronic devices especially in memory application.

#### 2.5.3 Sensing application

Sensor is a device, which provides direct information about the chemical composition of its environment [145]. In general a typical sensor device consists of a transducer and an active sensing layer. The active layer is suitably chosen in such a way that the interaction between the active layer and the sensing material (material to be sensed) will lead to a reasonable change in some physical properties. By observing the change in physical properties of interaction it will be possible to have idea about the target material. The combination of synthetic chemistry with the molecular engineering capability of the LB technique makes organic multilayered system interesting candidates for sensors. There are numerous physical properties based on which LB film based sensing system can be designed. Examples include resistivity changes, electro chemical phenomena, optical effects etc. Electrochemical pesticide sensor based on LB film of cobalt phthalocyanine-anthraquinone hybrid system has been demonstrated [146]. M. Ferreira et al. designed a sensor array by using nanostructured LB films for electronic tongue application. Such sensor was efficient to recognize sucrose, quinine, NaCl, and HCl at the parts-per-billion (ppb) level, being in some cases 3 orders of magnitude below the human threshold [147]. LB monolayers of tri-n-octylphosphine oxide-capped cadmium selenide quantum dots (QCdSe) onto indium−tin oxide (ITO) coated glass substrate was used to design an electrochemical DNA biosensor for detection of chronic myelogenous leukemia (CML) by covalently immobilizing the thiolterminated oligonucleotide probe sequence via a displacement reaction [148]. A H_2_ gas sensor has been demonstrated by using nanostructured PdO LB film which have high sensitivity over a wide range of concentration (30–4000 ppm) and fast recovery either on exposure to ambient light or by carrier gas flow. On exposure to ambient visible light the increase in photocurrent for such PdO films suggesting its plausible applications in solar energy conversion [149].

Many different types of organic materials have been used for LB film based gas sensing. These include porphyrins, phthalocyanines and insulating/conducting polymers [150, 151]. LB films of p-tert-butyl calyx (6) arene and calyx (6) arene showed organic vapour sensing properties [152]. LB films consisting of Fe_3_O_4_/γ-Fe_2_O_3_ nanoparticles have been used as the active layer in designing solid state resistive chemical sensor for selective sensing of NO_2_ and CO [153]. Hydrogen sensors based on Pd@Au core-shell nanoparticle has also been demonstrated [154]. To have a better understanding of the polymer/gas interactions electrochemical sensors (i.e. chemiresistors) have been widely investigated (also studies of mass and optical changes). Non-conductive polymers (i.e. those not containing π-bonds) have been used as mass sensors, thermal sensors, optical sensors and dielectric sensors [155, 156]. Polypyrrole (PPy) was widely used as various kinds of sensors depending on their transducing mechanism, including mass sensor [157], potentiometric sensor [158, 159, 160], potentiometric humidity sensor [157], amperometric sensor [161], amperometric biosensor [162, 163, 164], piezoresonance sensors [79] and conductometric sensor [[79], [165], [166], [167], [168], [169]].

**Fig. 4:**
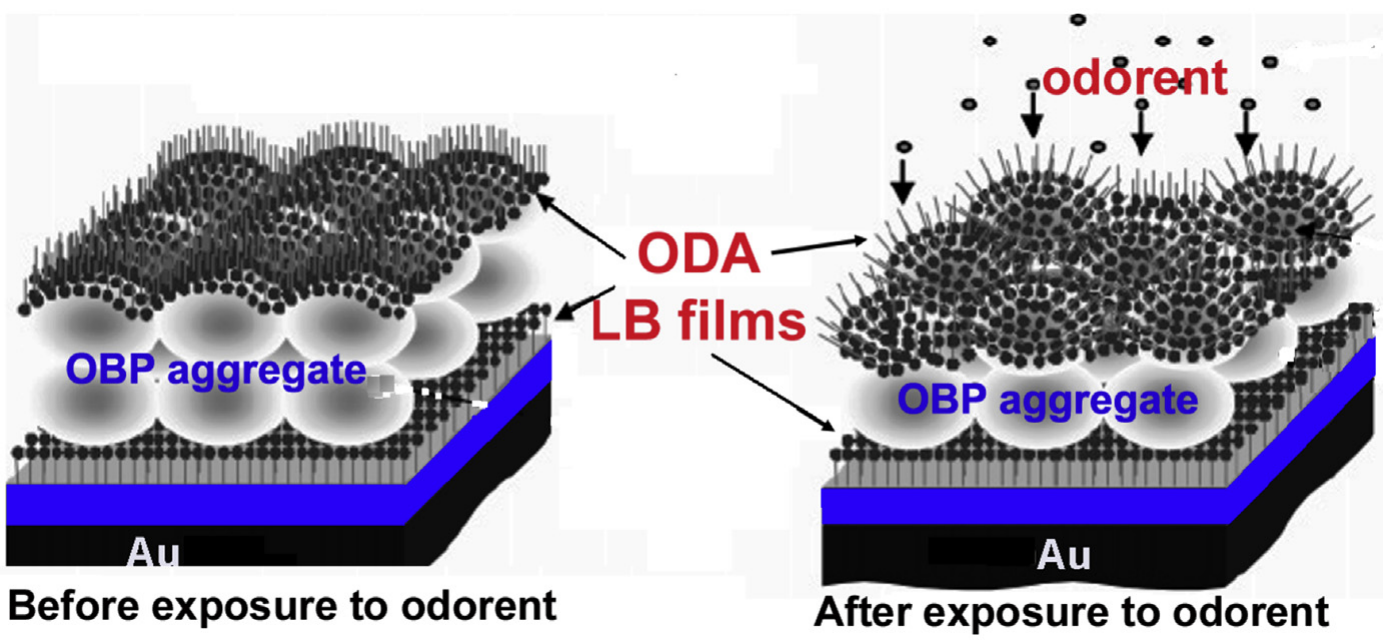
Schematic representation of LB film (OBP-1F/ODA) based odorant sensor [ODA: octadecylamine acetate & OBP-1F: Odorant-Binding Protein]. (Reprinted from Ref. [79], with permission from American Chemical Society).

Bio sensors are devises capable of retrieving analytical information from the operational environment by utilizing biological components as part of the sensor. Biosensors use biological molecules, mainly enzyme, lipids etc as the recognition elements. Professor Hou [79], demonstrated a novel biosensor using LB technique to sense the mixtures of odorants in various environmental conditions (see Fig. 4). LB method has been successfully used to mimic artificial biomembrane and to have idea about their thermodynamic behaviour as well as desiging FRET based cholesterol sensor [170].

**Fig. 5:**
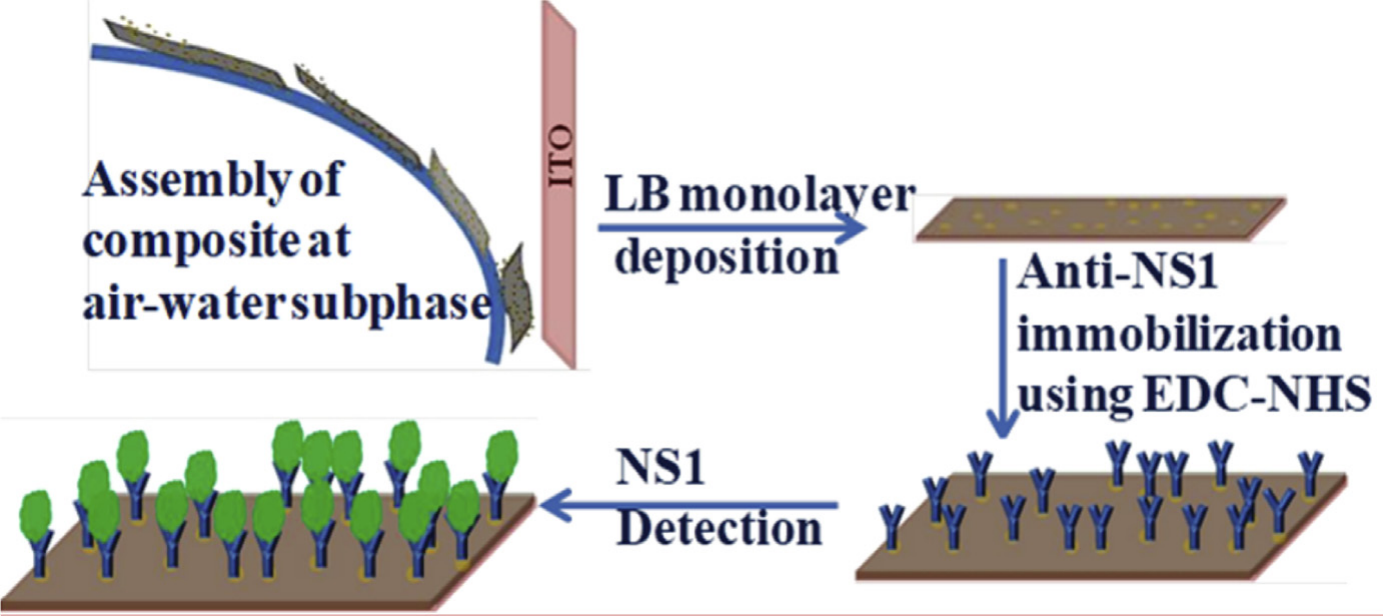
Schematic representation of MoS_2_−AuNP LB film based system for dengue detection (Reprinted from Ref. [173], with permission from American Chemical Society).

A novel optical nanosensor using a support bilayer lipid membrane (SBLM) has been recently proposed [171]. In this work LB and Layer-By-Layer (LBL) techniques have been combined to obtain highly ordered nanostructure. This work is particularly significant due to the importance of sensors for biological agents in vivo and/or in vitro. A heptamer linear RGD (acridine-glysine-asparate) containing peptide was covalently attached to a BODIPY (2-(4,4-difluoro-5, 7-diphenyl-4-bora-3a, 4a-diaza-s-dodecanoyl)-1-hexadecanoyl-glycero-3-phospho ethanolamine, donor) lipid dye and utilized as an optical biosensor. A second BODIPY (4,4-difluoro-5-(2-thienyl)-4-bora-3a,4a-diaza-s-indacene-3-dodec-anoic acid, acceptor) lipid dye was integrated into the SBLM, enabling the signal amplification via a Forster resonance energy transfer (FRET) mechanism. The result indicates the possibility to detect HUVEC at a concentration of 1000 cells ml^−1^. The sensitivity obtained by this method is similar to polymerized chain reaction (PCR) technique methods but less sensitive than flow cytometric techniques [114,172]. Biosensor for dengue detection has been demonstrated by utilizing LB films of molybdenum disulphide (MoS_2_) and gold nanoparticles (AuNPs). Here antibodies specific to dengue NS1 antigen has been immobilized onto thin films. The limit of detection (LOD) for such system is found to be suitable for clinical applications [173] (Fig. 5).

LB film based glucose sensor consisting of a conductive polypyrrole membrane has also been investigated [174]. Glucose sensing performance of glucose oxidase/Au nanoparticle composite assembled onto LB film has also been demonstrated [175]. Professor M. Rikukawa [176,177], fabricated a similar biosensor comprised of a lipid-modified glucose oxidase and conducting PPy LB film for detecting glucose. T. N. Misra, et al. demonstrated preparation of stable LB film of dehydrogenase (ADH) - stearic acid mixed system [178]. This mixed LB film has been deposited onto polypyrrole coated glass substrate. This LB-immobilized polypyrrole-mediated enzyme electrode can be used as an ethanol sensor. Novel enzymes based micrelectrochemical devices, based on changes in the conductivity of polypyrrole layers, were developed for biosensing of NADH and penicillin [179]. Enzyme catalytic properties have been enhanced by incorporating carbon nanotubes in penicillinase phospholipid Langmuir and Langmuir – Blodgett films [180]. Surface mediated drug delivery systems consisting of dendrons in presence and absence of a therapeutic compound in LB film has also been demonstrated [181].

Ordered ultrathin W_18_O_49_ nanowire assemblies have been used to prepare LB film based electrochromic devices [182]. the device display excellent stability when colour switching occurs, which may provide a versatile and promising platform for electrochromic device, smart windows and other applications [182].

Therefore, it is clear that LB technique showed enormous potential towards both fundamental research and device application. However, the key challenges to the researchers are the development of new sensing system and production of low cost, reproducible and reliable electronic devices with optimum sensitivities and selectivity.

## 3 Conclusions

Langmuir-Blodgett deposition technique is very simple but very powerful tool towards formation of nano dimensional ultrathin films with desired properties/architectures. Here each film is built from multiple monolayers. Accordingly the thickness and molecular arrangement is controllable at the molecular level. Using LB technique, it is possible to create complex yet precise artificial molecular arrays with predesigned physical and chemical properties. Already the potential of this versatile technique has been tested towards realization of optoelectronic devices. However, LB films have yet to be utilized in today's commercial products. The technique is still expected to be a vital part of future fabrication methods in molecular electronics. Unique dynamic nature of LB technique where Langmuir monolayer (typically one molecule thick) with large lateral dimension (2D) and molecular level control give us an opportunity for macroscopic action at molecular scale. This technique has the potential to build functional molecular system in a controlled manner at air-water interface and onto solid support, where they can be manipulated electrically or optically. This will open a new door to design solid state artificial molecular machines and devices [183]. Therefore, when molecular electronic and bio-electronic devices will become available, LB films will definitely play a vital role in their realization. However, commercial application of LB film is still lacking. Although extensive research work on various aspects of LB films are going on. Therefore main challenge to the researchers is demonstration of the commercial use of LB films. This need multidisciplinary outlook and collaborative efforts from almost all branches of science ranging from basic science to engineering Therefore it is highly appropriate to make a great stride in these important and promising areas of research, which can provide a conceptual understanding with wide opportunity of technological applications.

## Declarations

### Author contribution statement

All authors listed have significantly contributed to the development and the writing of this article.

### Funding statement

This work was supported by DST, Govt. of India (DST project Ref: EMR/2014/000234, FIST DST project Ref. SR/FST/PSI-191/2014) and by UGC, Govt. of India (UGC – SAP program 2016.)

### Competing interest statement

The authors declare no conflict of interest.

### Additional information

No additional information is available for this paper.
